# Deep Insights in Circular RNAs: from biogenesis to therapeutics

**DOI:** 10.1186/s12575-020-00122-8

**Published:** 2020-05-15

**Authors:** Peerzada Tajamul Mumtaz, Qamar Taban, Mashooq Ahmad Dar, Shabir Mir, Zulfkar ul Haq, Sajad Majeed Zargar, Riaz Ahmad Shah, Syed Mudasir Ahmad

**Affiliations:** 1 Division of Animal Biotechnology, Faculty of Veterinary Sciences and Animal Husbandry Shuhama, Sher-e- Kashmir University of Agricultural Sciences and Technology, Kashmir, 19006 India; 2 Department of Biochemistry, School of Life Sciences Jaipur National University, Jaipur, India; 3 Department of Biotechnology, University of Kashmir, Srinagar, India; 4 Division of Animal Breeding and Genetics, Faculty of Veterinary Sciences and Animal Husbandry, Shuhama, SKUAST-K, Srinagar, India; 5 Division of Livestock Production and Management, SKUAST-K, Srinagar, India; 6 Proteomics Laboratory, Division of Plant Biotechnology, Sher-e-Kashmir University of Agricultural Sciences & Technology of Kashmir, Shalimar, Srinagar, J&K 190025 India

**Keywords:** Circular RNA, Biogenesis, MicroRNA sponge, Gene expression regulation, Disease biomarker

## Abstract

**Abstract:**

Circular RNAs (circRNAs) have emerged as a universal novel class of eukaryotic non-coding RNA (ncRNA) molecules and are becoming a new research hotspot in RNA biology. They form a covalent loop without 5′ cap and 3′ tail, unlike their linear counterparts. Endogenous circRNAs in mammalian cells are abundantly conserved and discovered so far. In the biogenesis of circRNAs exonic, intronic, reverse complementary sequences or RNA-binding proteins (RBPs) play a very important role. Interestingly, the majority of them are highly conserved, stable, resistant to RNase R and show developmental-stage/tissue-specific expression. CircRNAs play multifunctional roles as microRNA (miRNA) sponges, regulators of transcription and post-transcription, parental gene expression and translation of proteins in various diseased conditions. Growing evidence shows that circRNAs play an important role in neurological disorders, atherosclerotic vascular disease, and cancer and potentially serve as diagnostic or predictive biomarkers due to its abundance in various biological samples. Here, we review the biogenesis, properties, functions, and impact of circRNAs on various diseases.

**Graphical Abstract:**

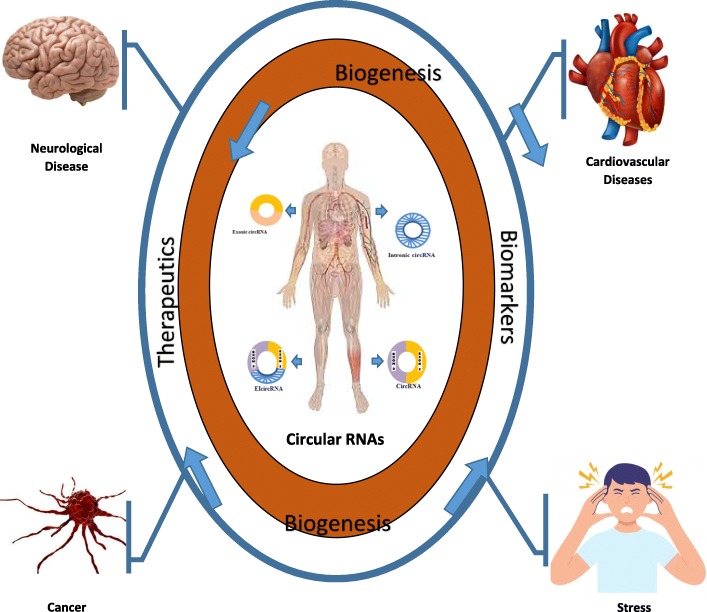

## Background

A new class of endogenous RNA (ceRNA) has come forth that forms a closed loop with no 5′-3′ polarities or poly-A tails [1], providing the stability and resistance to degradation by a variety of RNAase. CircRNA was initially identified from the transcript of a tumor suppressor gene [2]. Very few circRNAs were discovered in recent years [3–8]. These molecules were long considered as molecular flukes specific for certain pathogens; such as the hepatitis δ virus (HDV) [9] and some plant viruses [10]. However, this definition of “artifact” has recently been modified in the light of several recent studies revealing a significant amount of circRNAs present in different cell types of organisms extending from archaea to mammals. With recent advances in deep RNA sequencing technologies and bioinformatics, diverse properties were revealed and a large number of circRNAs like linear mRNAs were expressed in cells and tissues. For example, the first study to investigate circRNA-miRNA interaction revealed the loss of CD28 expression and circRNA100783 with phosphoprotein-associated functions during CD8(+) T cell ageing [11]. Exonic circRNAs are known to be identified from 14% of actively transcribed genes in human fibroblasts and 25,000 distinct RNA species containing a “back splice” reproduced by exonuclease degradation of linear RNA were validated as circular RNAs [12]. Mature circRNAs mainly are localized in the cytoplasm, but exon-intron circRNAs mainly appear to have nuclear origin due to their role in transcriptional regulation [13]. Its presence has also been validated in extracellular body fluids [cell-free saliva] of healthy individuals [14]. A majority of the circRNAs is synthesized endogenously as competing non-coding RNAs but a few exogenous circRNAs have been identified as hepatitis δ virus and genetically engineered circRNA with eukaryotic translational apparatus [15, 16]. Primarily they arise from exons, while some come from introns and have miRNA response elements (MRE). CircRNAs show least polymorphisms at the predicted miRNA target sites [17]. In addition to some circRNA, most sequences have been highly conserved evolutionarily between species [18]. So, overall the potential of circRNAs in transcriptional and posttranscriptional processes make them ideal biomarkers in the diagnosis of various diseases like cancer, neurological disorders, and cardiovascular diseases.

## Biogenesis

CircRNAs are pre-mRNA derived and their biogenesis during back splicing is spliceosomal or catalyzed by group I and II ribozymes [19]. However, the inhibition of canonic spliceosome reduces the levels of both circRNAs and linear transcripts, thus providing evidence of spliceosome involvement in the biogenesis of circRNA [20]. CircRNAs originate mainly from protein-coding exons but some from 3′-UTR, 5′-UTR, intronic and intergenic regions as well. CircRNA can be derived from both canonical and non-canonical cleavage processes. Different from the orthodox splicing of cognate linear mRNAs, circRNAs can originate from a single gene locus through alternative back-splice site selection and/or alternative splice site available in the CIRCpedia database [21].

So far, three types of circRNAs have been identified: exonic circRNAs (ecircRNAs), intronic RNAs (ciRNAs) [22] and exon-intron circRNAs (ElciRNAs). A study revealed 83% of circRNAs overlapping with protein-coding regions and exonic circRNAs account for the largest class found in animals and plants [23]. Some ecircRNAs can interact with microRNAs and/or RBPs, and many of them surround the other exon that contains canonical translation start codon [24].

### Lariat-Driven Circularization or Exon Skipping Model (Fig. 1a)

Exon skipping (ES) is a common type of alternative splicing with a well-known effect on mRNA formation [25]. A new study deciphered the important role that this process may have in ecircRNA biogenesis as well. During ES a large lariat containing the exon(s) is formed which subsequently undergoes internal cleavage to remove the intron and generate ecircRNA or EIciRNA [26]. The analysis of RNA Seq data sets has shown that the majority of the skipped exon(s) can produce ecircRNA in human umbilical vein endothelial cells stimulated by TGF-β or TNF-α [27]. In addition, exon production from ES is a very common step in the ecircRNA biogenesis of S. *pombe* [28]. Further studies are necessary to confirm if circularization proceeds with inherent properties of the lariat containing exon(s) or other factors such as RBP.

**Fig. 1:**
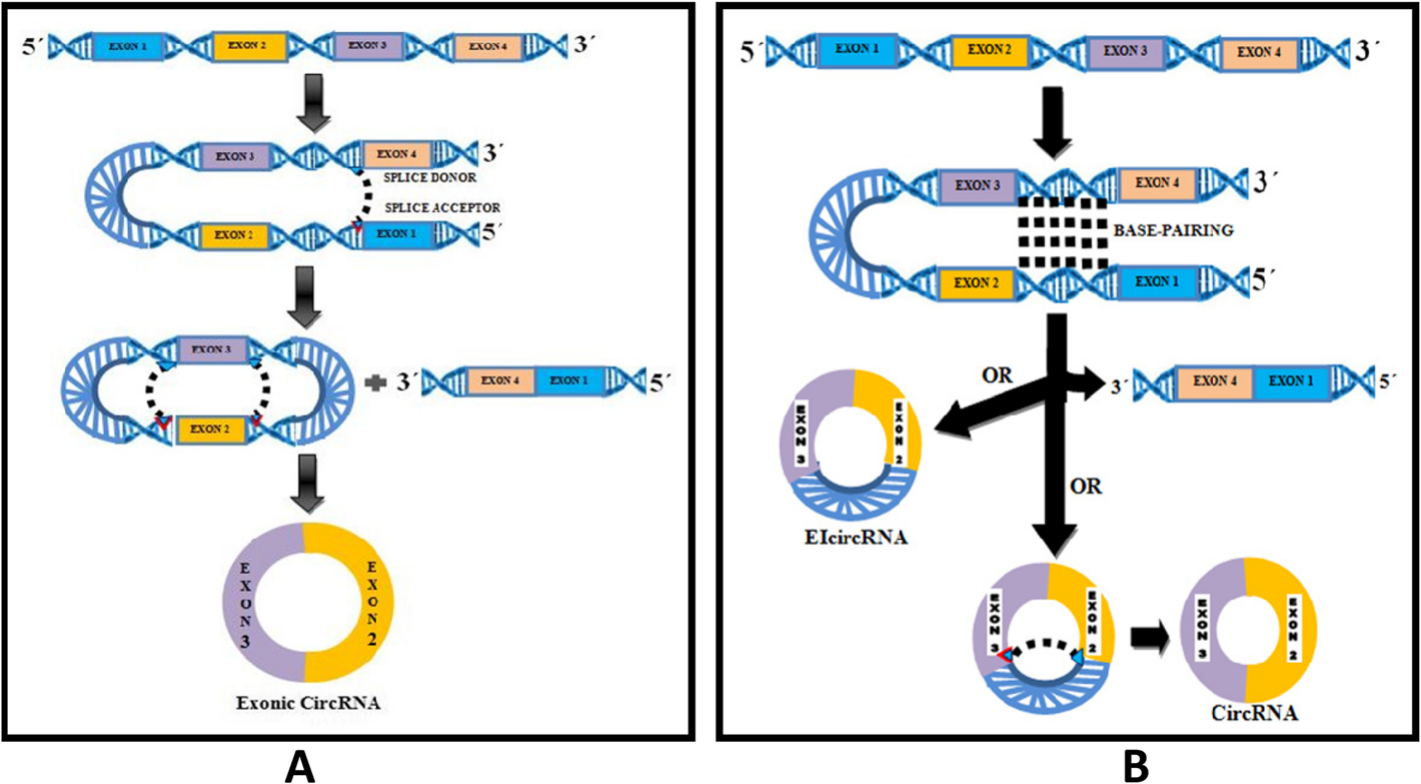
Models of circular Biogenesis **a**) Lariat-driven circularization or exon skipping model. Splice donor in 3′ end of Exon 1 covalently splices to splice acceptor in 5′ end of Exon 4 forms a lariat through Exon skipping. The Introns are removed through spliceosome to generate circular RNA. **b**) Intron-pairing driven circularization or direct back splicing model. Consecutive introns form a circular structure via base pairing. Introns are removed are reatained to form exonic circRNA or EIcircRNA

### Intron-Pairing Driven Circularization or Direct Back Splicing Model (Fig. 1b)

In direct splitting, exons are split in non-canonical order, a border point first attacks the downstream 5′-splice site (splice donor) at its 2′-hydroxyl group and then attacks the resulting 3′-hydroxyl end upstream of the 3′- splice site (splice acceptor) which forms a circRNA. It has been verified and suggested that cis-acting factors including the alternative form of inverted repeated Alu pairs (IRAlus) play role in multiple circRNA biogenesis [26, 29]. Genome-wide analysis in Drosophila has revealed that the lack of nucleotide motif for intron pairing in many gene loci produces abundant circRNA and the length of flanking introns appears to be a critical factor for back-splicing [30, 31]. CircRNA contains some repeated transposable sequences; such as Alu repeats and other short sequences, on their two flanking introns that form stable base pairs [32] to form a circular shape. After this, the spliceosome binds to the cyclic molecule under the effect of U6 and U2 and selectively cut the exons in the cycle region by interacting with the protein complex and the U5 nucleus [33, 34]. At the same time, exonic connections are reversed, forming mature circRNA. In addition, many circRNAs do not appear to have strong intron base pairs (62 and 91% in *C. elegans* and humans, respectively) [35]. Both the mechanisms of mammalian ecircRNA formation involve canonical spliceosome formation in-vivo, but as some linear mRNA lacks the exon to be incorporated in circRNAs, thus making intron paired circularization and not lariat-driven circularization likely for their biogenesis [36].

## Types of CircRNAs

### Intronic Circular RNAs (ciRNAs) (Figs. 2 & 3)

CircRNA derived from introns is known as circular intronic RNA (ciRNA). It was found to be abundant in the nucleus, reported firstly in human cells and was suggested to have a regulatory role in their parent coding genes. They represent a small part of circRNA, of which only 19.2% exists in humans [37]. and a very small fraction in plants. The mechanism of ciRNA formation differs from that of ecircRNA. Unlike ecircRNAs, the ciRNA has 2′-5′ head-tail joint, (Fig. 3) are not polyadenylated, distributed in the nucleus, associated with the nuclear insoluble fraction and are mainly less stable with few exceptions [38]. A consensus motif with a 7-nt GU-rich region near the 5′ splice site and an 11-nt C-rich region near branch point site are shown to be essential for an intron lariat to escape from debranching and formation of a circular structure. This is followed by the incision of exonic and intronic sequences in the binding portion of the spliceosome, and finally, the formation of mature circRNA as the residual introns are stitched together (Fig. 4) [39].

**Fig. 2:**
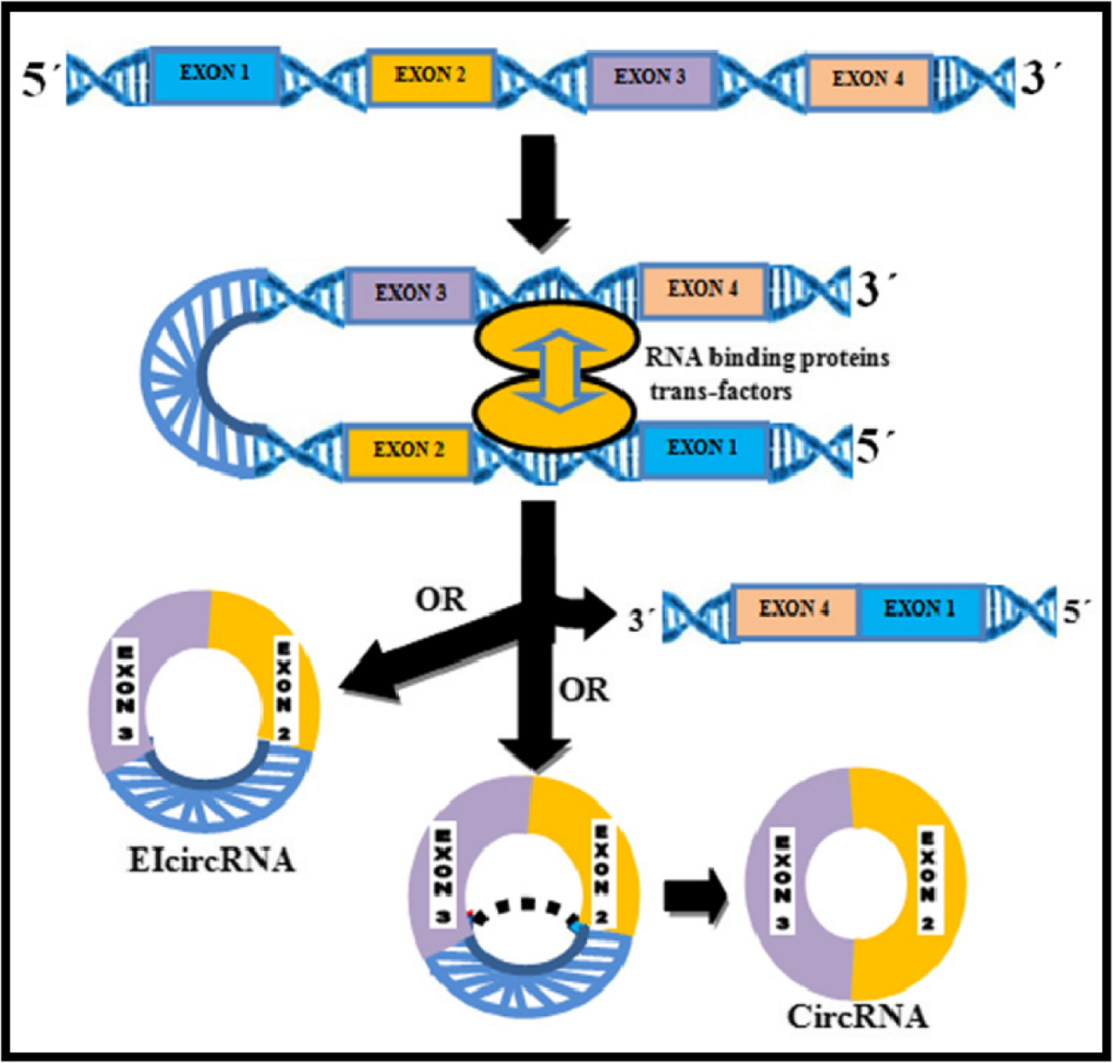
RBP or Trans Factor driven circularization. RBPs form a bridge between flanking introns by bringing splice donor and acceptor in close proximity to promote EcircRNA and EIcircRNA biogenesis

**Fig. 3:**
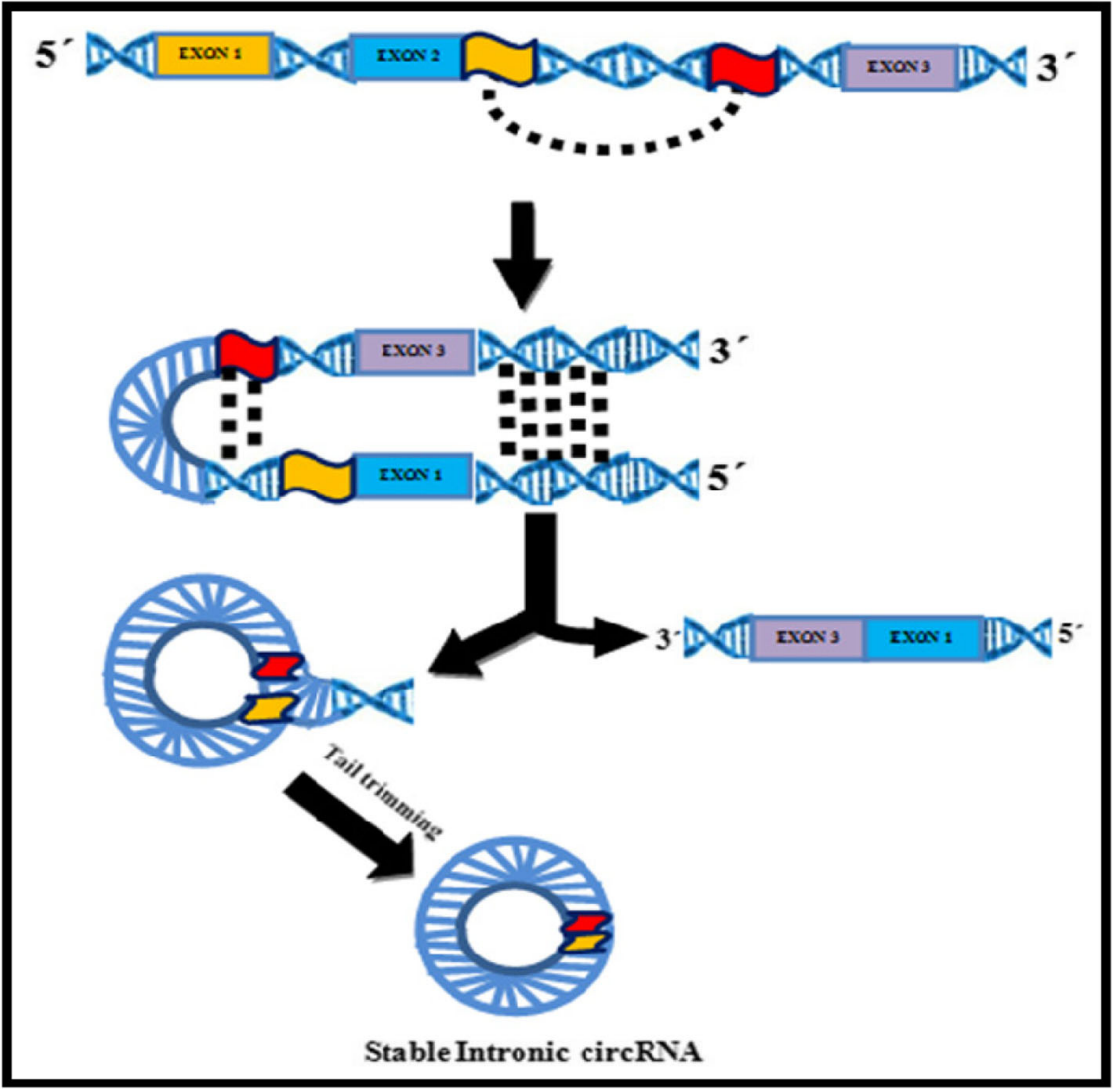
Circular Intronic RNA Biogenesis. Splicing reaction at GU rich sequences near 5′ splice site [RED) and C rich sequences near 3′ branch point [YELLOW) forms a lariat intron with 3′ tail downstream from the branch point to generate a stable Intronic circRNA

**Fig. 4:**
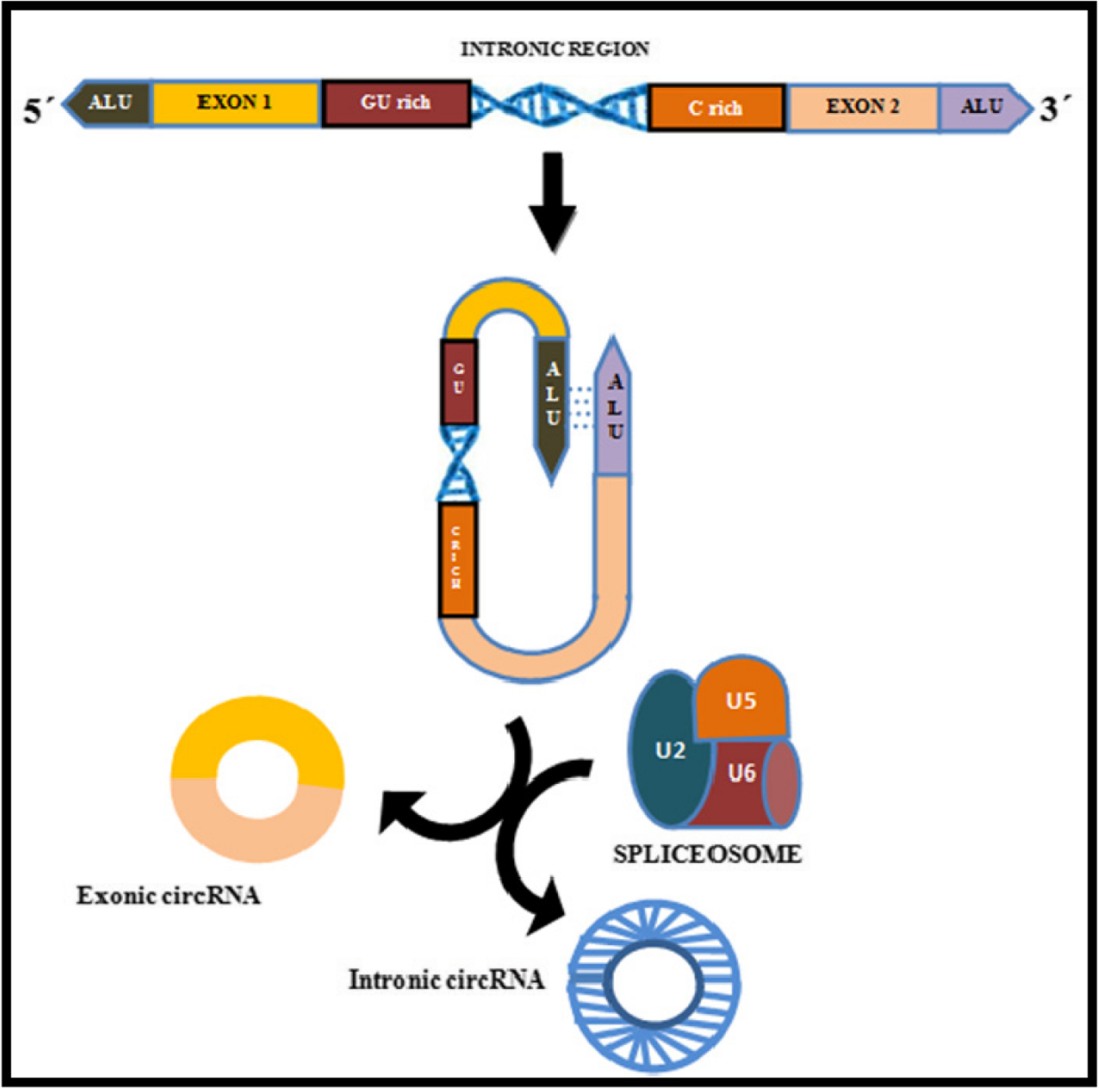
Different circRNAs have different back splicing mechanisms: Exonic circRNAs have cycling sequencing such as the ALU sequence, on the two sides, which binds to each other complimentarily. After that they are spliced by the spliceosome, which consists of U2 and U6. Intronic circRNA form a loop through the combination of upstream introns of the GU rich sequence and the downstream C rich sequence, and they are spliced by spliceosome

### Exon-Intron circRNAs (ElciRNAs) (Fig. 4)

A group of scientists found a novel type of circRNA where exons circulate together with introns, where the latter is “retained” between their exons and these are termed as exon-intron circRNA (EIciRNA). This unique subclass has the characteristics and functions of both exonic and intronic circRNAs. Similar to ecircRNA, EIciRNA’s complementary sequences are in their introns, indicating a similar mechanism of their biogenesis. Like ciRNAs, EIciRNA predominates in the nucleus. In RNA Polymerase II (Pol II) bound state it positively regulates the transcription of many parental genes by interaction with small nuclear ribonucleoprotein (snRNP) [13]. Direct back-splicing includes two pathways: First, intron pair-driven circularization and RBP pair-driven circularization that depends on sequences specific RNA-binding proteins and second exon- skipping that is essential to ensure proper production of circRNA. RBPs act as trans factors in regulating the activation or repression of both ciRNAs and EIciRNAs [40]. In fact, as the specific mechanism of the EIciRNA generation is unknown. Many ecircRNAs or ElciRNAs may arise from the same gene locus through alternative circularization. Recently, a study suggested a model of alternative splicing and back splicing for the complex biogenesis of multiple circRNAs in various cell line and further upgraded the CIRCexplorer2 pipeline (Fig. 2) [21]. Also, alternate splitting (AS) events within the circRNA of 10 human cell lines and 62 fruit fly samples were explored suggesting further studies on function and biogenesis of AS isoforms [41].

## Regulation

The regulation of circRNA biogenesis basically depends on the cis-regulatory elements and the trans-acting factors that govern splicing. Both the cis-elements and the trans factors promote circRNA biogenesis by getting a downstream donor and upstream acceptor sites in the vicinity. It has been found that complementary Alu repeats (cis-regulatory elements) are significantly enriched in circularized exons and introns flanking the circularized exons thus playing a role in RNA circularization in vitro and in vivo [12, 25, 35]. RBPs have been confirmed to serve a trans-regulatory role in the circRNA circularization, three of which have been identified. Muscleblind (MBL) a well-known splitting factor, binds to MBL binding sites on flanking introns of circular RNA (circMbl) and functions as a bridge for the circularization of its own second exon and circMbl formation [42]. Similarly, during epithelial to mesenchymal transition (EMT) another RBP, Quaking (QKI) belonging to STAR (signal transduction and activation of the RNA) family during cancer metastasis plays a role in circRNA biogenesis [43]. QKI affects mRNA turnover in various diseases including cancer [44] and for the generation of circRNA, there is a requirement for a specific RBP binding site within introns. Another regulatory enzyme is adenosine deaminase acting on RNA (ADAR-1), a double-stranded RNA editing enzyme, which negatively regulates the circRNA biogenesis. It has been proposed that ADAR1 promoted A to I editing that reduces pairing potential across Alu repeats in flanking introns. Its knockdown is shown to suppress circRNA formation [35]. Therefore, RBPs can act as activators or inhibitors for the formation of circRNA. Similar to linear RNA, the biogenesis of circRNA is also regulated by the spliceosomal RNA machinery. The RNA spliceosome is RNA-based enzyme with a U5 core consisting of 5 snRNA and several proteins. Under the effect of U6 and U2 promoters, the multiple proteins interact during the pre-RNA process [34]. In addition, a linear RNA molecule can be processed into different types of RNA, including mRNA, lncRNA, and circRNA, through various splicing events. For example, HIPK3 pre-mRNA can be divided into HIPK3 mRNA and its other exon can also form circRNA hsa_circ_0018082 [32]. Taken together, these shreds of evidence suggest that the biogenesis of the circRNA may depend on different factors that are likely to work together to regulate the results of back splicing. In addition, different circRNAs can be regulated with different mechanisms and their production in cells seems to be more complicated than previously believed.

## CircRNA Transportation and Stability

Circular RNAs have long half-lives because they are naturally resistant to RNA exonuclease degradation due to their unique covalent bond between the 5 ‘and 3’ ends. This high stability suggests that the progressive accumulation of circRNA is dominated by their slow turnover instead of the production, resulting in accumulation in the cells. Two mechanisms were suggested for their transport; firstly, using the exon junction complex to assist in export from the core [45] and another is the secretion of circRNA from cells via extracellular vehicles (EVs). EVs are membrane-bound vessels delivered from the cells and may contain cellular components, including proteins, lipids, and RNAs. On the basis of their biogenesis and pathways, EVs are characterized, exosomes being a specific type of EVs. Also, it was demonstrated that exo-circRNAs within serum exosomes of colorectal cancer (CRC) patients represent a novel class of stable RNAs. The expression level of circKLDHC10 was found to be significantly higher in CRC patients than in healthy individuals. Also, the accumulation of circRNA during neuronal differentiation, synaptic development, and foetus development has been observed. Therefore, the excess of circRNA in body fluids and blood due to high RNAase resistance allows them to serve as potential biomarkers in various diseases. Nevertheless, cellular levels of circRNAs are highly controlled; endonucleases provide access points for the exonuclease, probably by facilitating the disintegration of circRNAs [46]. The main RNA endonucleases in eukaryotic cells include Argonaute 2 (Ago-2) (which works in RNA silencing), Angiogenin (which cleaves tRNA during stress), CPSF73 (which works in the formation of mRNA 3 end), IRE1 (which works in ER stress), RNAase L (which is involved in native immunity) and SMG6 (which is important for non-sense mediated breakdown). One of the circRNA detected in EVs include ciRS-7/CDR1as, which indicate EVs mediated transport of circRNA via cell-to-cell communication. The CDR1as / ciRS-7 contains an almost perfect miR-671 target site that can be cleaved by Ago-2 to trigger the degradation of the transcript. Possibly, the packaging of circulating RNAs in EVs is either to eliminate excess circular RNA or involvement in cell-to-cell communications.

## Functions

### CircRNAs as miRNA Sponges

Accumulated evidence indicates circRNAs to act as potent miRNA sponges or competing ceRNA molecules, but since the circRNA has no free ends, these are predicted to avoid microRNA mediated deadenylation [47]. CeRNA molecules such as mRNA, lncRNA, and pseudogenes, contain shared microRNA response elements that allow competition for miRNA binding [48] suggesting its role in miRNA functioning and regulating gene expression. The strongest evidence of the sponging activity of the circRNA is derived from a study of exonic circRNA, ciRS-7/ CDR1ase, which sponges mR-7 or CDR1 antisense and murine sex-determining region Y (Sry). Both ciRS-7 and Sry are known to bind miRNAs without degradation, making them powerful ceRNAs. Testis-specific murine Sry gene (sex-determining region Y) responsible for sex determination in many mice species was shown to produce a single exon circular transcript found freely in the cytoplasm [5] that serves as the miR-138 sponge. Transfection studies in HEK293 cells have shown that co-transfection with Sry, vector, and pJEBB-138, this single-exon circRNA can sponge miR-138 and can accumulate Argonaute (AGO) proteins in a miR-7-dependent manner [24]. Circular antisense transcript (CDR1as), the translational product of the cerebellar degeneration-related protein 1 (CDR1) gene cannot be degraded by RNA-induced silencing complexes (RISCs) [24] but seems to be degraded by miR-671 but not miR7 [49]. The binding site of miR-671 shows a little variation across species and possesses near-perfect complementarity. The altered expression of CDR1as and its inhibitor miR-671 alters the expression of genes such as an alpha-synuclein gene (SNCA), epidermal growth factor receptor (EGFR) gene and Insulin receptor substrate 2 (IRS2) [50, 51]. Bioinformatics analysis of mammalian ecircRNA data generated by circRNA-Seq experiments revealed very few circRNAs with more than 10 miRNA binding sites. For example, circ-ITCH is known to span several exonic binding sites of ITCH gene and acts as a miRNA sponge of miR-7, miR-17, and miR-214. In contrast to circRNAs in mammals, *D. melanogaster* circRNAs possess at least one thousand well-conserved miRNA seed matches [30] but fly circRNAs functioning as miRNA sponges are yet to be elucidated. Overall, whether sponging activities of circRNAs is a general phenomenon or not and how the balance between networks of circRNAs, miRNAs, and ceRNAs is maintained to regulate cellular homeostasis is still a mystery.

### CircRNAs as Regulators of Alternative Splicing, mRNA Trap, and Transcriptional Regulations

CircRNAs are regulators of alternative splicing and transcription. CircMbl, generated by the second exon of a splicing factor MBL competes with the pre-mRNA splicing process. CircMbl has conserved sites on the flanking introns that strongly and specifically bind MBL. Therefore, MBL may regulate alternative splicing by modulating the balance between canonical splicing and circMbl biogenesis. CircMbl as mRNA traps act to regulate translation by sequestering the translation start site [52]. Mouse formin (Fmn) gene for limb development can produce exonic circRNA via backsplicing of the Fmn coding region. Moreover, the exonic circRNA decreases the expression level of the Fmn protein [53]. It was further uncovered in human fibroblasts that 34% of the circRNAs with a single exon contain a translation start site [36]. Exonic circRNAs derived from HIPK2 and HIPK3 loci undergo alternative splicing rather than canonical splicing for circularization to produce a protein-coding transcript that is conserved between mice and humans [12]. Their functioning as mRNA traps is of great importance as they can regulate the phenotypic effect of any target gene. For example, in dystrophinopathy patients, the mRNA trapping by Dystrophin exonic circular RNAs might enhance the disease phenotype leading to inactive DMD transcripts, further reducing the pool of translatable mRNAs [54]. However, emerging therapies for these dystrophinopathies that regulate splicing events are presently in clinical trials [55].

### CircRNAs as Regulators of Parental Gene Expression

As demonstrated in Fig. 3 circRNAs act as regulators of parental gene expression. CircRNA biogenesis is mainly dependent on intronic lariat formation because they have less affinity for microRNA target sites, indicating their distinctive functionality. Some circRNAs located in the nucleus of human cells, CircEIF3J and CircPAIP2 require Pol II to modulate host-transcription in a cis-acting manner. They interact with the U1 subunit of small nuclear penteconucleotides thereby acting in a cis mode to improve parent gene transcription. Likewise, Li and others revealed that both circ-ITCH and its 3′-UTR share some miRNA binding sites for miR-7, miR-17, and miR-214, which increases the expression of ITCH. Thus it is speculated that intrinsic circRNAs such as ciRNA and EIciRNA, as opposed to ecircRNA can function effectively in cytoplasmic regulatory processes that seem to predominantly regulating the transcription process in the nucleus.

### Translation Potential of CircRNAs

Lack of essential structures critical to efficient translation initiation regarded circRNAs with no protein-coding ability, but researchers were propelled to search for the translational potential of circRNAs due to its origin from protein-coding regions, open reading frames (ORFs) and cap-independent translation of linear mRNAs. Naturally occurring circRNA in mammalian cells with protein-coding ability was primarily “hepatitis δ agent” [9]. Early in 1995, an in vitro study revealed mammalian translation apparatus could initiate translation of engineered circRNAs [16]. When IRES gets inserted into a green fluorescent protein (GFP), the resulting circRNA serves as mRNA directing robust GFP protein synthesis [31]. Subsequently, a circRNA of a virusoid (220 nt in length) associated with the rice yellow mottle virus (Sobemovirus) generates a 16-kD protein [56]. The circRNA database named circRNADb provides [57] detailed information about the genomic sequences, IRE sites, and ORFs etc. of human circRNAs. There are no experimental shreds of evidence to prove that ecircRNAs serve as mRNAs. Also, no naturally occurring ecircRNA that undergoes translation (i.e., bound to polysomes) has been discovered so far [58]. Unlike linear products of genes that were significantly abundant in the ribosome-bound fractions, no circular species of these genes were bound to monosome or polysomes. Ribosome footprinting data for human bone osteosarcoma epithelial cells (U20S) also does not support the natural existence of circRNAs that undergo translation [59]. It has been recently reported that the most common base modification of RNA [N6-methyladenosine (m6A)] promoted efficient protein translational initiation from circRNAs in human cells. Additionally, the consensus N6-methyladenosine (m6A) motifs enriched in circRNAs drive translational initiation. Initiation factor eIF4G2 is required which is further enhanced and inhibited by methyltransferase METTL3/1 and demethylase FTO respectively [60]. Polysomes profiling analysis with mass spectrometry and computational prediction revealed the widespread translational potential of hundreds of endogenous circRNAs.

## CircRNAs in Cancer

The high stable expression and presence of circRNAs in human body fluids, such as serum, plasma, saliva, and exosomes in significantly higher quantities make circRNAs as ideal biomarkers for cancer [61]. CircRNAs are dysregulated and linked with the upholding of various cancer phenotypes. The expression of various circRNAs is dysregulated in esophageal squamous cell carcinoma (ESCC), for instance, circ- ITCH, hsa_circ_000167, hsa_circ_001059 and hsa_circ_0067934. Circ-ITCH aids in antitumor activity by participating in downregulating c-myc oncogene and ubiquitin-mediated degradation of Dvl2 [62]. RNA-Seq data from patients with colorectal cancer (CRC) tissues and normal colon, revealed the global reduction of circRNAs in patients with CRC [63] as well as presence of circRNAs in serum exosomes of CRC patients and not in healthy controls. Similarly, in gastric cancer (GC) tissues the significant lower expression of hsa_circ_002059 than in healthy controls was known to be associated with, gender, TNM stage, age and metastasis making it a new biomarker for gastric cancer [64]. In laryngeal squamous cell cancer (LSCC) tissues, microarray data revealed differential expression of circRNAs (302 upregulated and 396 downregulated circRNAs), hsa_circRNA_100855 being the most upregulated circRNA [65]. In hepatocellular carcinoma (HCC), overexpression of circZKSCAN1 in vivo and in vitro is known to inhibit cancer proliferation and metastasis but was found to be significantly lower in cancerous tissues than in healthy controls [66]. Similarly in HCC prognosis, Circ-ZEB1.33 serves as an important biomarker as it was overexpressed in HCC tissues and serum samples compared to non-tumorous tissues and in healthy control serum samples while its levels are correlated with TNM stages and overall survival in HCC patients. On the other hand, in breast cancer, ESRP1/circANKS1B/miR-148a/ 152-3p/USF1 regulatory circuit contributes to breast cancer invasion and metastasis and induce epithelial to mesenchymal transition through activating the TGF-β1 signaling pathway, thereby, facilitating the development of new treatment strategy against the metastasis of breast cancer [67]. CircRNAs are abundantly present and specifically regulated in breast cancer as shown by RNA Seq data. In primary breast cancers, circRNAs of CREBBP, CNOT2, and RERE and RNAi-mediated knockdown of circRNA circCNOT2 was shown to significantly reduce the viability of two breast cancer cell lines: MCF-7 and BT-474 [68]. Hsa_circ_0008039 might play a potential tumor-suppressive role and act as a therapeutic target for breast cancer treatment. It promotes proliferation, cell cycle progression, and migration and contributes to malignant behaviors in breast cancer while sponging the activity of miR-432-5p via enhancing E2F3 expression [69]. Mechanistic analysis indicates the overexpression of circRNA-000911 not only suppressed the proliferative and metastatic capacity of the breast cancer cells but also promoted the apoptosis of cancer cells by specifically sponging miR-449a, releasing Notch1 and promoting the NF-κB signaling pathway. Thus it may serve as a potential predictive therapeutic breast cancer biomarker [70]. In GC tissues, the expression of hsa-circ 0000745 was an indicator of tumor differentiation, while as its expression in plasma correlated with tumor metastasis [71]. In Oral squamous cell carcinoma (OSCC), hsa_circ_0001874 and hsa_circ_0001971 serve as OSCC diagnostic biomarkers among many upregulated and downregulated circRNAs as compared to healthy controls [72]. While as in urothelial carcinoma, circPRMT5 acts as a prognostic biomarker as its higher level in both serum and urine correlates with tumor progression and lymph node metastasis [73]. So with more research on the role of circRNAs in cancer, their importance in many other cancers in overexpressed as well as under-expressed conditions will be elucidated in the near future.

## CircRNAs act as miRNA Sponges in Cancer

CircRNAs reduce the expression of miRNA-mediated gene regulation in cancer types. This interaction with tumor-related miRNAs indicates a great significance in tumor biology. Gene ontology (GO) enrichment analysis from 174 human disease-associated miRNA data including cancer concludes circRNAs to interact with disease-associated miRNAs. A breakthrough study reported circRNAs to function as miRNA sponges, which are known to naturally sequester and inhibit their target miRNA activity [74]. CircRNAs recognize and bind target miRNA on complementary sequences between the seed region (2–7 nts in the mature miRNA sequence). Mutations in miRNAs seed regions and target sites have a high impact on the miRNA–ceRNA interactions [75]. The dysregulation of crosstalk between miRNAs and ceRNAs significantly affect cancer pathogenesis, suggesting a correlation with miRNAs as well as the involvement of circRNAs in malignant tumors. For example, the ciRS- 7/miR-7 axis is involved in cancer via down-regulating gene expression of oncogenes like XIAP and EGFR. Tumor suppressor genes such as KLF4 are also inhibited by the same axis, which promotes gastric cancer, cervical cancer, hepatocellular carcinoma, schwannoma tumor, tongue cancer, lung neoplasm, and CRC by sequestering and inhibiting miR-7 activity [76, 77]. Furthermore, miR-7 upregulate E cadherin that reduces EMT thereby promoting the conversion of highly invasive breast cancer cells with mesenchymal characteristics to the cells with epithelial properties [78]. Dysregulation of Wnt/ β-catenin signaling pathway by cir-ITCH is considered a widespread theme of cancer biology. Additionally, E6/E7 a viral oncogene is known to overexpress miR-7 activity in HPV-positive human HeLa cells [79]. Also, Cir-ITCH has been reported sponging various miRNAs such as miR-20a and miR-7 in CRC and miR-7, miR-17, and miR- 214 in esophageal squamous cell carcinoma. Some circRNAs like circ-ZEB1.19, circZEB-1.17, circZEB1.5, and circZEB1.33 suppress lung cancer progression by acting as miR-200 sponge target for ZEB1. CircRNAs are abundantly conserved over various species and have tissue and developmental stage-specific properties. In essence, they are strongly expressed in blood, saliva and circulating exosomes, serving as essential cancer-specific biomarkers for the diagnosis, prognosis, and therapy. Upregulation of circRNA_100855 and downregulation of circRNA_104912 in LSCC is significantly correlated with stage and metastasis. Many circRNAs are known to express themselves in various human cancers including hepatocellular carcinoma and colorectal carcinoma [80–82] indicating the critical role in physiological and pathological processes of cancer. In summary, these findings suggest significant biological roles of the circRNA in developmental processes and the ability to act as potential new biomarkers in various cancers.

## CircRNAs in Neurological Diseases

The abundance of circRNAs and differential expression in various regions of brains and neuronal tissues such as cortex, olfactory cortex, hippocampus, striatum and cerebellum show their upregulation during neuronal differentiation and role in neuronal functions [83]. The abundance of circRNAs in neuronal tissues like post-mitotic neuron is also contributed by its higher stability. RNA Seq studies have shown plenty of circRNAs in synaptic segments of neurons where they encrypt synaptic proteins and that the circRNAs reside in both dendrites and cell body [84]. The natural occurrence of circRNAs in mammalian cells makes them more prone to be expressed in both natural and diseased conditions and in the later case the circRNAs are significantly expressed in the midbrain region and various disorders related to nervous system such as Parkinson’s disease (PD), Alzheimer’s disease (AD), multiple sclerosis (MS), and schizophrenia (Table 1) [85]. Alzheimer Disease is one of most dreadful neurological disorders associated with downregulation of number of miRNAs and there is a deficiency in the levels of Ubiquitin-Conjugating Enzyme (UBE2A) an enzyme essential for the proteolytic clearance of AD-amyloid peptides in human CNS. One of an abundant and well-conserved miRNA found in human brains and murine CNS is miR-7 [86] that is sponged naturally by ciRS-7. In AD brain sponging activity of ciRS-7 on miRNA- trafficking down-regulates the expression of the UBE2A and EGFR [87]. However, upregulation of miR- 7 may suppress AD-relevant targets suggesting that CDR1as (inhibitor of ciRS-7) is involved in AD pathogenesis. Moreover, the expression reports of circRNAs in 5 and 10-month old senescence accelerated mice P8 (SAMP8) mice were identified using circRNA microarray and found 85 dysregulated circRNAs in 10 months old SAMP8 versus control mice and 231 circRNAs exhibited differential expression in 10 months old SAMP8 versus 5 months old SAMP8. Thus providing a valuable resource for the diagnosis and therapy of AD [88]. Microarray analysis technology provided novel mechanisms underlying AD as well as novel therapeutics by characterizing the expression patterns of circRNAs, miRNAs and mRNAs in hippocampal tissue from Aβ_1_–_42_-induced AD model rats [89]. In PD brains, the accumulation of alpha-synuclein in the Lewy bodies within brain nigral neuronal cells is prevented by the activity of miR-7 which downregulates alpha-synuclein are protects the cells against oxidative stress [50]. So the inhibition of miR-7 by CiRS-7/CDR1as is involved in PD pathogenesis. There are several other miRNAs that on downregulation are involved in PD such as let-7, miR-153 and miR-34a/b [90–92]. The microarray based circRNA expression profile of plasma in systemic lupus erythematosus (SLE) patients and normal participants reveal four circRNAs, hsa: circ_102584, hsa: circ_400011, has: circ_101471 and hsa: circ_100226 to be dysregulated in SLE plasma and suggests the role of circRNAs as potential biomarkers in SLE [93]. In ALS (Amyotrophic lateral sclerosis), an RNA binding protein (FUS) is a new regulator of controlling circRNA expression in mouse motor neurons and controls the splicing of novel and unknown transcripts. Its link to circRNA function and regulation in neurodegenerative processes may lead to new understanding into the mechanism of mutant FUS-associated ALS and related disorders [94]. Some circRNAs are also involved in diverse inflammatory processes due to their abundance and presence on immune cells like circRNAs hsa 2149 is found in leukocytes but absent from neutrophils, or HEK293 cells. Biogenesis of circRNA 100,783 involves splicing-related mechanisms during CD28-related CD8 (+) T cell ageing and senescence. In neuropathy, suppression of a transcription factor, RUNX3 I in an autoimmune disease by miR-138 could balance the expression of CD4+ T cells [95]. CircRNAs are abundantly expressed in various cell line and a study on glioblastoma cell line demonstrated that the suppressed activity of miR-7 via its inhibitor CDR1as downregulated IRS-1 and IRS-2 expression by reducing the PK-B activity and hence acts as a therapeutic target for malignant gliomas [96]. A study reported for the first time circRNAs from MS associated locus. Expression of circRNA, hsa_circ_0043813 from the STAT3 gene was shown to be associated with disease associated SNPs hence suggesting role of circRNAs as novel contributors in MS pathogenesis [97]. However, much more study on the functions and structure of circRNAs is needed that would provide a platform for utilizing novel technologies and broaden our insight regarding the disease pathogenesis and for diagnostic and treatment purposes.

**Table 1:** List of circRNAs related to Neurological Diseases

Neurological Disease	Circular RNA	Targets	Mechanisms	References
**Inflammatory Neuropathy**	hsa-circRNA 2149	miR-138	Specific expression in leukocytes Involved in chronic CD28 associated CD8^+ T^ cell aging.	Fu et al.*,* 2015
	CircRNA hsa 2149			
			Balance the expression between Th1 and Th2 through suppressing the function of RUNX3.	
**Alzheimer’s Disease**	CDR1as	miR-7	Downregulate AD relevant targets, such as UBE2A, which plays an important role in clearance of amyloid peptides in AD.	Zhao et al., 2016 Lukiw et al.*,* 2016
**Nervous system Neoplasms**	CDR1-as	miR-7	Represses the expression of EGFR, IRS1, & IRS2. Thereby reducing the active and aggressive glioblastoma.	Liu et al., 2014
**Parkinson’s Disease**	CDR1-as	miR-7	Downregulate α-synuclein expression and protect cells against oxidative stress.	Junn et al.*,* 2009
**Amyotrophic lateral sclerosis**	Known and Unknown CircRNAs	FUS	FUS regulates biogenesis of CircRNAs in mouse Motor Neurons.	Errichelli et al.*,* 2017
**Systemic lupus erythematosus**	hsa: circ_102584,	(hsa-miR-766-3p, hsa-miR-762, hsa-miR-412-3p, hsa-let-7i-3p and hsa- miR-431-3p)	Mechanism not clear	Haixia et al.*,* 2018
	hsa: circ_400011,	(hsa-miR-296-3p, hsa-miR-146b-3p, hsa-miR-181d-3p, hsa-miR-504-3p and hsa-miR-328-5p)		
	hsa: circ_101471	(hsa-miR-136-5p, hsa-miR-665, hsa-miR-486-3p, hsa-miR-601 and hsa-miR-30b-3p)		
	hsa: circ_100226	(hsa-miR-138-5p, hsa-miR-145-3p, hsa-miR-24-3p, hsa-miR-620 and hsa-miR-875-3p)		

## CircRNA in Cardiovascular Diseases

In cardiovascular diseases circRNAs play very important roles that has been summarized in (Table 2). One of an important heart related circRNA is HRCR which sponges the activity of miR-223 as well as its downstream target ARC (apoptosis repressor with CARD domain) thus preventing the induction of heart related problems like cardiac hypertrophy, heart failure and hypertrophy in cardiomyocytes [98]. However, one of an inducer of myocardial Infarction (MI) is CDR1as that sponges the activity of miR-7. Myocardial apoptosis due to prolonged ischemia results from Cdr1as/miR-7a axle through induction of PARP and SP1 but are regulated negatively via CDR1as and thus preventing the myocardial cells from MI injuries during hypoxia [99–101]. Another important circRNA is circ-Foxo3, which exhibits pro-senescence function due to a direct circ-Foxo3 and senescence relationship as the former downregulates cell growth and cell cycle progression [102] and hampers the transport of antisenescence transcription factors into the cell nucleus. In cardiac tissue circ-Amotl1 potentiates AKT enhanced cardiomyocyte survival by phosphorylation and nuclear translocation of AKT1. Also, in vivo, circ-Amotl1 had a protective effect against induced cardiomyopathy [103]. Human microarray correlated expression of hsa_circ_0003575 to oxLDL induced HUVECs proliferation and angiogenesis. It was confirmed through silencing of hsa_circ_0003575, which would provide a therapeutic strategy for endothelial cell injury in atherosclerosis [104]. Also in HUVECs, knockdown studies and luciferase assay on hsa_circ_0010729 revealed its physiological role in cardiovascular system disease thus identifying its role on vascular endothelial cell proliferation, migration and apoptosis via targeting miR- 186/HIF-1a axis [105]. Genome wide Association studies (GWAS) have revealed the correlation between small nucleotide polymorphisms (SNPs) near INK4/ARF locus and Arteriosclerotic Vascular Disease (ASVD) [106]. SNPs regulate transcription through modulating cANRIL production, [107] which further modulate atherosclerosis susceptibility. cANRIL is a useful susceptibility marker to ASVD and promotes antiatherogenic cell functions due to its stability against degradation [108, 109]. It prevents key mechanisms in atherosclerosis by inducing apoptotic pathways via inducing nucleolar stress and inhibits proliferation by activation of p53, a suppressor of cell cycle [110]. In mice model of diabetic myocardial fibrosis, a novel circRNA circRNA_010567 was found to promote myocardial fibrosis. Its sponging activity was elucidated through RNAi mechanism when knockdown of circRNA_010567 upregulated miR-141 and downregulated TGF- β1. As a miR-141 sponge it suppresses fibrosis-associated proteins in cardiac fibroblasts like Col I, α-SMA and Col III and directly affects the expression of TGF- β1 [111]. In a study, blood samples were collected from coronary artery disease (CAD) patients and healthy individuals for screening of circRNAs. Microarray analysis compared the circRNA profile and revealed 22 blood circRNAs to be differentially expressed between the two groups. Since the aim of the study was to look for a potential diagnostic biomarker for CAD, hsa_circ_0124644 was selected after following a stringent screening criteria as GO revealed its role in variety of processes like apoptosis etc. [112]. Also, in a study with 40 differentially expressed circRNAs in blood of control and experimental groups, only one circRNA, hsa -circRNA11783–2 was closely related to patients with both CAD and type 2 diabetes mellitus (T2DM) rather than in patients suffering from CAD and T2DM alone [113]. Deep RNA sequencing revealed top highly expressed circRNA corresponding cardiac genes including Titin, RYR2 and DMD and most of the cardiac expressed circRNAs were heart specific [114].

**Table 2:** List of circRNAs related to Cardiovascular Diseases

Disease Type	CircRNA	Targets	Effect on Diseases	Ref.
**Heart failure and pathological hypertrophy**	HRCR	mmiR-223, ARC	Suppression	Wang et al., 2016
**Myocardial Infarction**	Cdr1as (ciRS-7)	miR-7, SP1, PARP	Induction	Geng et al.*,* 2016
**Cardiac senescence**	Circ-Foxo3	ID1,E2F1,FAK,HIF1a	Suppression	Du et al.*,* 2017
**Atherosclerosis**	cANRIL	INK4/ARF locus	Regulation	Song et al.*,* 2017
	hsa_circ_0003575	miR-199-3p, miR-9-5p, miR-377-3p and miR-141-3p	Regulates oxLDL induced vascular endothelial cells proliferation and angiogenesis	Li et al.*,* 2017
	hsa_circ_0010729	miR-186/HIF-1α axis	Regulates vascular endothelial cells proliferation and apoptosis	Dang et al.*,* 2017
**Cardiomyopathy**	Circ-Amotl1	AKT1	Regulation	Zeng et al.*,* 2017
**Myocardial Fibrosis**	CircRNA_010567	miR-141, TGF- β1, Col I, α-SMA and Col III	Promotes Fibrosis	Zhou et al.*,* 2017
**Coronary artery disease**	hsa_circ_0124644	–	Biomarker	Zhao et al., 2017
	hsa -circRNA11783–2	–	No functional role elucidated	

## CircRNA in Stress and Senescence

CircRNA profiles in aged human peripheral blood not only provide epidemiological evidence for a role in human aging phenotypes, or lifespan, but also in vitro evidence that some circRNA may influence cell senescence phenotypes [115]. In stress related pathways, circ-FOXO3 plays an important role as its dysregulation results in sequestration of various proteins. While as its role in cardiac senescence is marked by it induction in doxorubicin treated mice and ROX treated mouse fibroblasts and interaction with E2F1, HIF-1a, FAK and ID-1, stress pathway related proteins [102]. As stress is one of the causative agents for neurological diseases, the targeting of circRNAs produced by Homeodomain-interacting protein kinase-2 and 3 (HIPK-2 and 3) enzymes that are mostly stimulated during genotoxic stress may be effective for patients with stress [116]. Microbial infections generate acute stress responses that are rapid in nature and cause activation of various signaling pathways like NF-κB signaling pathway that are involved in activation of stress related transcriptional factors. LPS induces various circRNAs, and one of such is circ-RasGEF1B that is known to be involved in fine-tuning immune responses on RAW264.7 cells in association with various TLRs. It is known to be cell type specific and conserved between mouse and humans. Its function was elucidated by knockdown and was shown to regulates ICAM-1 expression, wherein it decreased the stability of mature mRNA [117]. The expression arrays of circRNAs in early passage and senescent (late passage) human diploid WI-38 fibroblasts were compared with RNA Sequencing and senescence-associated circRNAs (SAC-RNAs) were identified. CircPVT1 was found to have a correlation with senescence as it was elevated in dividing cells by modulating and sequestering let-7 activity to enable a proliferative phenotype [118]. In another case whole transcriptome sequencing enabled the identification of SACs in young and prematurely senescent human diploid fibroblast 2BS cells. A circRNA CircCCNB1 was found to have a role in delaying cellular senescence by sponging miR-449a. Also it can represent a promising strategy for age-related disease interventions [119].

## CircRNA-Based Therapeutic Strategies

Circular RNAs emerge as potential therapeutic tools due to their ability to regulate gene expression. Various molecular-based manipulation tools are currently under investigation. Also, to understand the impact of altered expression of circRNAs, functional characterization is necessary, which is usually assessed through circRNA overexpression, and/or knockdown experiments. RNA interference is one of the easiest yet challenging methods compared to other RNA types in which loss-of-function experiments determine the functional relevance of circRNAs. siRNAs are synthesized to target few base pairs with complementarity only at the backsplice junction while leaving the linear RNA unaffected. Thus, siRNA-mediated knockdown approaches severely restrict to the backsplice junction, and negative effects on the parental protein-coding gene expression are always tested for and ruled out. Silencing of circCCDC66 in colorectal cell lines demonstrated the oncogenic role of this circRNA in various cancer-related processes like proliferation, invasion, migration and anchorage independence. It acts as a sponge to a number of oncogenes and prevents them from degradation that was proved by manipulating the levels of circCCDC66. Many of oncogenes were downregulated once the circCCDC66 was silenced one of them being MYC that was extensively studied [120]. In HCC tissues and cell lines, circZKSCAN1 was found to be significantly lower and knockdown promoted cell proliferation, migration and invasion in both in vitro and in vivo [66]. While as in GC, hsa_circ_0000096 levels were significantly downregulated in gastric cancer tissues and gastric cancer cell lines and its knockdown inhibited cell proliferation and migration in vitro and in vivo [121]. However, the major concern with RNAi is the off target effects which lead to unanticipated consequences like gene suppression that need to be carefully avoided [122]. As naked single-stranded RNA strands are highly vulnerable and prone to nucleolytic degradation. Thus, for in vivo experiments, chemically modified antisense oligonucleotides (ASOs) such as multiple “second generation” GapmeRs could be use with higher stability [123]. These can be used as an alternative to target the primary structure of circRNAs. GapmeRs are commonly used to target lncRNAs so it is conceivable that they may additonally downregulate circRNAs in vivo, which is yet to be confirmed [124]. However, in a study by Zhang et al., ASOs were designed to target intronic circRNA [22] as well as to the back-splicing junction in the EIciRNA [13]. (CRISPR/Cas9) Clustered regularly interspaced short palindromic repeats-associated nuclease Cas9, can function efficiently delete circRNAs either partially or totally without hampering the functions of linear coding mRNA. In a study CRISPR-Cas9 technology was used to knockout the locus encoding Cdr1as. It is a cytoplasmic circRNA that is highly expressed in neurons. CDR1as loss-of-function mutant mice were generated. But mostly genome editing based knockout of circRNAs involve deletions of circRNA exons, which is difficult as CRISPR/Cas9 off target effects can’t be ruled [125]. Zhang et al. described that genome editing of the intronic RNA pairing through approach of CRISPR/Cas9 specifically knocks out the expression of a circRNA expression without affecting its residential linear mRNA. Editing the intronic complement sequence (ICS) of the circGCN1L1-flanking introns, circGCN1L1 expression was abolished that was sufficient to remove its expression without effecting the linear mRNA expression from the GCN1L1 gene locus [126]. So, CRISPR/Cas9 technology offers an unprecedented prospect for fractional or whole knockdown of circRNAs involved in cancer. Also, RNA-guided RNA-targeting CRISPR-Cas effector (Cas13a) also known as C2c2 was engineered as a new tool for RNA modulation, for RNA knockdown in mammalian cell and for studying RNA in mammalian cells. The most effective is Cas13a from Leptotrichia wadei (LwaCas13a) that is expressed in both plant and mammalian cells. It targets knockdown of both reporter as well as endogenous transcripts. When compared to RNAi, knockdown efficiency of LwaCas13a is comparable with high specificity, efficiency and flexible RNA targeting [127]. Moreover, the inactive and catalytically dead variant (dCas13) binds to programmed and targeted RNA molecules and protein domain like adenosine deaminase domain of ADAR2 [128]. Overexpression of circRNA can be achieved by easily manipulable circRNA overexpression plasmids containing the circRNA sequence. Unlike mammalian genes, whole genes can be expressed on a plasmid. Mammalian vectors only contain the circRNA sequence flanked by splicing signals, intronic sequences and harbor inverted repeats. However, in this case RNA polymerase will continue to transcribe around the entire plasmid if it fails to recognize the transcription terminator site (TTS), generating a concatemer of the RNA sequence contained in the plasmid that results in a number of undesired transcripts [129]. To rule out these concatemer, checking the overexpression product size is necessary. The overexpression construct can be delivered by plasmid transfection [24] or viral vector systems like adenovirus vectors [98] or Adeno associated virus AAV vectors [130]. However, new delivery strategies avoiding viral vectors have been a great deal of interest due to many advantages. One of the non-viral RNA delivery vehicles is the nanoparticle encapsulation of RNA, which physically protects it from degradation. Another method is to conjugate a bioactive ligand to the RNA that will allow it to enter the cell of interest [131]. In another approach for stable protein expression in eukaryotes, exogenous circRNAs were produced through in vitro transcription (IVT) and purified through HPLC. These stably transfected circRNAs produce more quantity of proteins than modified linear mRNA or unmodified counterparts. However, for future in vivo approaches, suitable delivery strategies are needed [132].

## Conclusion

CircRNAs are proved to be the key players in the emergence of diseases, such as cancer, cardiovascular, neurological diseases and many more. The function of these molecules is under ambiguity as far their expression in various diseased conditions is concerned. Due to their higher structural stability, circRNAs regulate many metabolic processes, have characteristics to serve as diagnostic or predictive biological markers of various dreadful diseases and can serve as new possible therapeutic targets. Nevertheless, there is still some gaps left in our understanding of circRNAs, as their intricate molecular mechanisms in the development of various diseases is yet to be fully annotated. But the dawn of research and technical advances, such as the development of RNAseq and bioinformatics approaches to detect circRNAs would explain the functions of circRNAs in terms of pathological and physiological processes in the future and this precious world of circRNAs can bestow upon us with new insights into the “dark matter” of the genome. Functional experiments, such as knockdown of expression of specific circRNAs, would determine how circRNAs regulate cellular pathways and determine specific biomarkers that would help in the early disease diagnosis, high-risk population identification, assess response and finally to develop targeted therapies. Though circRNA based research is early, due to their tissue specificity, stability, and suitability in "liquid biopsies it may provide the next generation of personalized medicine and continued exploration into circRNAs will help us to better understand it’s heterogeneous and dynamic aspects.

## Data Availability

All the data supporting the results are included in the article.

## Abbreviations

CircRNAs: Circular RNAs
ncRNA: non-coding RNA
miRNA: microRNA
RBPs: RNA-binding proteins
HDV: hepatitis δ virus
MRE: miRNA response elements
ElciRNAs: exon-intron circRNAs
ecircRNAs: exonic circRNAs
ciRNAs: intronic RNAs
ES: Exon skipping
IRAlus: Inverted repeated Alu pairs
snRNP: small nuclear ribonucleoprotein
AS: alternate splitting
EMT: epithelial-to-mesenchymal transition
MBL: Muscleblind
QKI: Quaking
STAR: signal transduction and activation of the RNA
ADAR-1: adenosine deaminase acting on RNA
EVs: extracellular vehicles
CRC: colorectal cancer
ceRNA: endogenous RNA
Sry: sex-determining region Y
CRD1: cerebellar degeneration-related protein 1
CDR1as: Circular antisense transcript
AGO: Argonaute
SNCA: alpha-synuclein gene
EGFR: epidermal growth factor receptor
IRS2: Insulin receptor substrate 2 gene
Fmn: formin
Pol II: RNA polymerase II
ORFs: open reading frames
GFP: green fluorescent protein
ESCC: esophageal squamous cell carcinoma
OSCC: Oral squamous cell carcinoma
GC: Gastric cancer
LSCC: laryngeal squamous cell cancer
fcircRNA: fusion circRNA
GO: Gene ontology
PD: Parkinson’s disease
AD: Alzheimer’s disease
MS: multiple sclerosis
CNS: central nervous system
ALS: Amyotrophic lateral sclerosis
GWAS: Genome wide Association studies
APT1: acyl protein thioesterase 1
UBE2A: Ubiquitin Conjugating Enzyme
APT1: acyl protein thioesterase 1
ARC: apoptosis repressor with CARD domain
ASVD: Arteriosclerotic Vascular Disease
MI: Myocardial Infarction
CAD: coronary artery disease
HIPK-2: Homeodomain-interacting protein kinase-2
HIPK3: Homeodomain-interacting protein kinase-3
SAC-RNAs: senescence-associated circRNAs
ASOs: antisense oligonucleotides
CRISPR/Cas9: Clustered regularly interspaced short palindromic repeats-associated nuclease Cas9
